# Synergistic Effects of Ammonia and Hypoxia Stress on the Transcriptomic Responses of the Razor Clam (*Sinonovacula constricta*)

**DOI:** 10.3390/ani16060896

**Published:** 2026-03-12

**Authors:** Zidai Liu, Hao Zhang, Congying Lai, Ran Sun, Hongqiang Xu, Hanhan Yao, Yinghui Dong, Zhihua Lin, Liyuan Lv

**Affiliations:** 1 Ninghai Institute of Mariculture Breeding and Seed Industry, Zhejiang Wanli University, Ningbo 315100, China; coral08111@163.com (Z.L.); 17815953320@163.com (H.Z.); 18318798169@163.com (C.L.); sun1003750230@163.com (R.S.); xuhongqiang@zwu.edu.cn (H.X.); zhihua9988@126.com (Z.L.); 2 Zhejiang Key Laboratory of Aquatic Germplasm Resources, College of Biological & Environmental Sciences, Zhejiang Wanli University, Ningbo 315100, China; yaohanhan@zwu.edu.cn; 3 College of Advanced Agricultural Sciences, Zhejiang Wanli University, Ningbo 315100, China; dongyinghui118@126.com

**Keywords:** *Sinonovacula constricta*, ammonia, hypoxia, combined stress, arginine metabolism, HIF signaling, organ-specific response

## Abstract

The co-occurrence of ammonia nitrogen and hypoxia represents a physiologically taxing synergistic challenge for benthic bivalves—as it forces a conflict between the high energy demand for detoxification and the limited energy supply under low oxygen, yet the tissue-specific strategies underlying their resilience remain poorly understood. This study investigated the physiological and transcriptomic responses of the razor clam *Sinonovacula constricta* to ammonia (AG), hypoxia (HG), and their combination (HAG) over 96 h. Transcriptomic profiling revealed that the gill and hepatopancreas employ distinct, organ-coordinated adaptive strategies rather than a uniform systemic response. The gill prioritized respiratory homeostasis by fine-tuning oxygen sensing: transcriptional suppression of hypoxia-inducible factor 1-α (*HIF-1α*) (to limit glycolytic acidosis) was followed by a chronic induction of *HIF-2α*, alongside the specific upregulation of the mitochondrial respiratory gene cytochrome c oxidase-6b (*COX-6b*). In contrast, the hepatopancreas executed a critical metabolic trade-off centered on arginine metabolism. Under combined stress, arginine flux was redirected toward the urea cycle via a robust upregulation of arginase (*ARG*) for detoxification, while nitric oxide synthase (*NOS*) was concurrently suppressed. This reciprocal regulation suggests a strategy to prioritize ammonia clearance and energy conservation at the expense of immune signaling. These findings elucidate how *S. constricta* navigates the bioenergetic conflict between detoxification and oxygen limitation, providing molecular targets for breeding stress-resistant aquaculture strains.

## Simple Summary

In contemporary intensive aquaculture, the razor clam frequently encounters the dual threat of high ammonia nitrogen and low dissolved oxygen (hypoxia). This study identifies how different tissues in the clam coordinate their molecular “defensive shields” to survive these stressors over 96 h (the 96 h exposure period was selected based on its widespread application in acute stress studies of *S. constricta*. This duration is a well-established physiological window that allows for the observation of stable metabolic shifts and tissue-specific adaptive responses to ammonia and hypoxia, without reaching critical mortality levels that would compromise the integrity of the omics analysis.). We discovered that the clam does not respond uniformly; instead, the gill and hepatopancreas perform specialized roles. The gill maintains respiratory health by switching its oxygen-sensing machinery—temporarily suppressing *HIF-1α* to avoid metabolic acidosis and later activating *HIF-2α* along with the energy-producing gene *COX-6b*. Simultaneously, the hepatopancreas acts as a detoxification hub, prioritizing the clearance of poisonous ammonia through the urea cycle (via *ARG* upregulation) at the expense of its immune signaling (*NOS* suppression). This strategic trade-off reveals how the razor clam balances its limited energy between self-cleansing and survival under environmental pressure. These findings provide critical molecular markers for selecting and breeding more resilient clam strains for sustainable aquaculture.

## 1. Introduction

The razor clam, *Sinonovacula constricta*, constitutes a dominant component of mariculture in China and Southeast Asia. It is distinguished by its rapid growth rate [1,2,3] and high nutritional value and significant economic returns, with an annual production exceeding 800,000 tons [4]. Unlike pelagic species, *S. constricta* is a typical benthic burrowing, filter-feeding bivalve inhabiting the sediment–water interface of intertidal mudflats, which exposes the clams to drastic environmental fluctuations [5]. In recent decades, the intensification of high-density aquaculture, coupled with global warming and coastal eutrophication, has severely deteriorated sediment quality. These conditions frequently lead to the simultaneous occurrence of ammonia accumulation and dissolved oxygen depletion (hypoxia), particularly during summer or following algal blooms [6]. Consequently, deciphering the physiological resilience of *S. constricta* to these complex stressors has become a critical priority for industrial sustainability.

Ammonia nitrogen [6,7,8] and dissolved oxygen [9,10] are two pivotal abiotic factors governing the survival and homeostasis of aquatic ectotherms. Ammonia, the primary end-product of protein catabolism in bivalves, acts as a potent toxicant. Excessive accumulation leads to gill hyperplasia, neurotoxicity, and systemic oxidative stress by disrupting the antioxidant defense system [11]. Concurrently, oxygen is imperative for aerobic metabolism. Hypoxia not only restricts the ATP supply required for basal maintenance but also impairs the energy-dependent excretion of nitrogenous wastes. This creates a bioenergetic conflict imposed by the high energy demands of active ammonium transport and glutamine synthesis [12]. Consequently, hypoxia potentially exacerbates ammonia toxicity by inhibiting the ATP supply required for detoxification, leading to metabolic acidosis and mortality [9,13].

Unlike teleosts that rely heavily on branchial excretion, benthic bivalves have evolved unique metabolic strategies to survive in oxygen-poor and ammonia-rich sediments. A key adaptive mechanism involves metabolic depression, a dormancy-like state where ATP turnover is actively suppressed to extend survival [7]. Furthermore, bivalves possess high plasticity in nitrogen metabolism. Rather than relying solely on passive diffusion, certain bivalves can modulate nitrogen metabolism pathways, such as glutamine synthesis [14], or potentially utilize specific amino acids like arginine as a metabolic buffer. Arginine, in particular, plays a pivotal dual role by functioning as both a substrate for the urea cycle to facilitate detoxification and a precursor for nitric oxide (NO), a key regulator of vascular tone and oxidative stress response [15]. However, whether this arginine-mediated regulation functions as a central coping mechanism under the specific context of combined ammonia and hypoxia stress in *S. constricta* remains to be verified.

While the independent effects of ammonia and hypoxia are well-documented, these stressors rarely occur in isolation in aquatic environments. Current research, however, has largely overlooked the complex biochemical ‘cross-talk’ that occurs when these two stressors interact, particularly the specific ways different organs—such as the gills and hepatopancreas—prioritize metabolic functions to survive this dual threat. Studies in teleosts and crustaceans have confirmed that the combined stress of ammonia and hypoxia (HAG) exerts a synergistic toxicity exceeding the sum of individual effects [16,17,18]. Specifically, hypoxia compromises the ATP-dependent transport for ammonia excretion, while ammonia-induced tissue damage further reduce respiratory efficiency [19]. In vertebrate models such as *Micropterus salmoides* [17] and Hybrid groupers [18,20], this synergistic impact is characterized by a synchronized upregulation of oxidative, apoptotic, and inflammatory pathways. Physiologically, this is manifested by initial increases in antioxidant enzyme activities (SOD, CAT, GSH-Px) followed by their depletion, alongside significant accumulation of malondialdehyde (MDA) and lactic acid. At the molecular level, dual stress upregulates a broad spectrum of genes associated with antioxidants (e.g., *HSP70*, *HSP90*), apoptosis (*p53*, *Bax*, *Caspases*), and inflammation (*TNF-α*, *IL-1β*), indicating a compounded burden of cellular damage. Metabolically, the organism attempts to compensate by enhancing anaerobic capacity, as evidenced by the upregulation of key glycolytic genes (*GLUT1*, *MCT1*, *PFK*, *LDH*). In summary, dual stress exerted a significantly greater effect on the organism than single stress, with its synergistic action exacerbating the physiological burden and metabolic disruption.

In contrast to the extensive data on vertebrates, our understanding of these synergistic mechanisms remains fragmentary for benthic mollusks, particularly intertidal bivalves like *S. constricta*. These soft-bodied animals primarily inhabit the infaunal zone, living buried within seafloor sediments. Unlike mobile species that can migrate to avoid unfavorable conditions, these mollusks possess limited mobility, making them highly susceptible to localized environmental stressors. This vulnerability is exacerbated by global climate change; as water temperatures rise, the solubility of oxygen decreases while the microbial decomposition of organic matter in the sediment accelerates, leading to the simultaneous occurrence of hypoxia and the accumulation of toxic ammonia [5]. Consequently, these organisms are frequently trapped in a “double-stress” environment within the sediment–water interface. Current knowledge is largely confined to single-stressor responses. Regarding ammonia stress, *S. constricta* and other aquatic species are known to employ strategies such as enhanced autophagy, immune activation, and specifically, the upregulation of glutamine synthesis and urea cycle genes to facilitate detoxification [14,21]. Similarly, under hypoxic stress, bivalves initiate adaptive metabolic reprogramming regulated by the HIF signaling pathway to transition from aerobic to anaerobic metabolism [22,23]. However, it remains unclear whether these distinct adaptive strategies function synergistically or antagonistically when *S. constricta* confronts both stressors simultaneously. Specifically, the tissue-specific molecular strategies employed by organs such as the gill (central to respiration and immunity) and the hepatopancreas (the hub of metabolism and detoxification) to coordinate a systemic response under dual stress have yet to be fully elucidated.

To address this gap, this study employed high-throughput transcriptomics integrated with Weighted Gene Co-expression Network Analysis (WGCNA) to characterize the dynamic responses of gill and hepatopancreas in *S. constricta* under ammonia, hypoxia, and their combination. Following this study, we aimed to: (1) characterize the temporal shifts in gene expression profiles under single and combined stressors; (2) elucidate the tissue-specific adaptive mechanisms, focusing on the switch in oxygen sensing pathways and the reprogramming of arginine-mediated nitrogen metabolism; and (3) identify key regulatory modules serving as potential biomarkers. These findings provide a comprehensive molecular framework for understanding how benthic bivalves navigate complex environmental challenges.

## 2. Materials and Methods

### 2.1. Experimental Animals and Stress Treatments

Adult razor clams, *S. constricta* (shell length of 65.43 ± 4.02 mm, range: 57.45–73.12 mm; wet weight of 15.19 ± 3.22 g, range: 9.15–22.5 g), were obtained from Ninghai Institute of Mariculture Breeding and Seed Industry in June 2024. Prior to experimentation, the clams were acclimated for five days in 25 L tanks containing filtered seawater (temperature 20–21 °C and salinity 22–23 ppt) with continuous aeration. Clams were fed *Chlorella* sp. Daily, and the water was renewed entirely every day.

**Figure A1:**
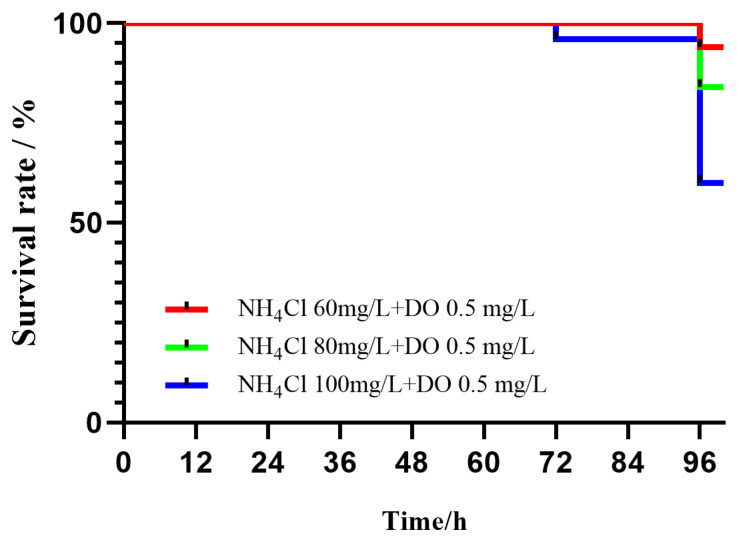
Screening of ammonia nitrogen concentration gradients in combined ammonia nitrogen and hypoxia stress pre-experiment.

Based on preliminary trials and a 96 h acute toxicity test (which determined the LC50 of ammonia for *S. constricta* to be approximately 100 mg/L under a hypoxic background of 0.5 mg/L O_2_ [24], Appendix A Figure A1), clams were randomly assigned to four experimental groups with three biological replicates each. The detailed information of the four experimental groups were: control group (CG): maintained in normoxic seawater with standard aeration; ammonia nitrogen stress group (AG): exposed to 100 mg/L total ammonia nitrogen (added as NH_4_Cl) under normoxic conditions; hypoxia stress group (HG): exposed to 0.5 mg/L dissolved oxygen (DO)—hypoxia was achieved and maintained by nitrogen gas injection into a sealed system [24]; the combined stress group (HAG): exposed to 100 mg/L NH_4_Cl and 0.5 mg/L DO simultaneously. Water temperature and salinity were maintained consistently with acclimation conditions. To maintain stable ammonia concentrations and hypoxia levels, water was renewed every 12 h, and reagents (NH_4_Cl) or N_2_ gas were replenished immediately. Gill and hepatopancreas tissues were dissected from 20 individuals per replicate at 0 h, 6 h, and 96 h. Samples were immediately frozen in liquid nitrogen and stored at −80 °C for subsequent RNA extraction.

### 2.2. Transcriptomics Analysis

#### 2.2.1. RNA-Seq Library Preparation and Sequencing

Total RNA was extracted from gill and hepatopancreas tissues using VAHTS Universal V10 RNA-seq Library Preparation Kit (Vazyme, Nanjing, China) following the manufacturer’s protocol. RNA integrity and concentration were assessed using 1% agarose gel electrophoresis and an Agilent 2100 Bioanalyzer (Agilent Technologies, Santa Clara, CA, USA). Only samples with an RNA integrity number (RIN) ≥ 7.0 and total amount ≥ 800 μg were used for library construction. A total of 42 cDNA libraries (2 tissues × 7 conditions × 3 replicates) were constructed. Poly(A)-tailed mRNA was enriched, fragmented, and reverse-transcribed into cDNA. Libraries were size-selected, PCR-amplified and sequenced on the Illumina NovaSeq X Plus platform (Illumina, San Diego, CA, USA) to generate 150 bp paired-end reads.

#### 2.2.2. Bioinformatics Analysis

Raw reads were processed using fastp (v0.23.2) to remove adapters and low-quality sequences. Clean reads were aligned to the *S. constricta* reference genome [2] using HISAT2 (v2.2.1). Transcript assembly was performed with StringTie (v2.2.1), and novel transcript prediction were annotated using NR, Uniprot, KEGG, and GO databases. Gene expression levels were quantified using featureCounts (v2.0.1) and normalized as Fragments Per Kilobase of transcript per Million mapped reads (FPKM). Differentially expressed genes (DEGs) were identified using DESeq2 (v1.16.1) with a threshold of |log_2_Fold Change| ≥ 1 and an adjusted *p*-value < 0.05. Functional enrichment analysis of Gene Ontology (GO) and Kyoto Encyclopedia of Genes and Genomes (KEGG) pathway were performed on DEGs using clusterProfiler package (v4.2.0) in R (v4.1.3).

#### 2.2.3. Weighted Gene Co-Expression Network Analysis (WGCNA)

To elucidate the molecular signatures underlying the response of *S. constricta* gill and hepatopancreas to ammonia and hypoxic stress, co-expression networks were constructed using the WGCNA package (v1.73) in R (v4.5.1). Genes with low expression variance (SD ≤ 0.5) were filtered out. Raw expression data were pre-filtered to exclude lowly expressed transcripts (RPKM < 1). Subsequently, hierarchical clustering of samples was performed to detect and remove potential outliers, ensuring high data consistency. A soft-thresholding power (*β*) was selected (β = 28 for gill; β = 20 for hepatopancreas) to satisfy the scale-free topology criterion. For the gill tissue, β = 28 was chosen as the point where the scale-free fit index (R^2^) entered a plateau (reaching 0.7), ensuring a balance between network sparseness and biological connectivity. For the hepatopancreas, the index successfully reached the standard threshold of R^2^ > 0.8 at β = 20. The detailed power selection analysis is provided in Appendix A Figure A4. Finally, genes were clustered into color-coded modules based on topological overlap measures (TOM) dissimilarity. Module eigengenes (MEs) were correlated with stress conditions (ammonia, hypoxia, and combined stress) to identify stress-responsive modules.

### 2.3. Quantitative Real-Time PCR (qRT-PCR) Validation

Five representative genes were selected for validation via qRT-PCR. These genes included arginase (*ARG*), a pivotal enzyme in ammonia detoxification via the urea cycle; nitric oxide synthase (*NOS*), which regulates nitric oxide (NO) production; cytochrome c oxidase (*COX*), which is involved in oxidative phosphorylation; and *HIF-1α* and *HIF-2α*, which are involved in the hypoxia response pathway.

Primers were designed using Primer Premier 5.0 (Table 1). Total RNA (1 µg) was reverse-transcribed using the HiScript III RT SuperMix (Vazyme, Nanjing, China). qRT-PCR were performed on a CFX Opus Deepwell Real-Time PCR System (Bio-Rad, Hercules, CA, USA) using ChamQ Universal SYBR qPCR Master Mix (Vazyme). The thermal cycling conditions were: 95 °C for 30 s, followed by 39 cycles of 95 °C for 10 s and 59 °C for 30 s. The reaction system comprised a total volume of 10 μL, with 5 μL of 64-fold diluted cDNA template, 0.5 μL of each primers (10 µmol/L), and 4 μL of ChamQ Universal SYBR qPCR Master Mix (Vazyme). The *RS9* gene was used as the internal reference. The stability of *RS9* in *S. constricta* has been previously validated [25], and its consistent Ct values (variation < 24 cycles) across our treatment groups further confirmed its suitability for this study. The relative gene expression levels were calculated using the 2^−ΔΔCt^ method.

All data are presented as means ± standard error of the mean (SEM). Statistical analyses were performed using IBM SPSS Statistics 22.0. Differences between the control group and each treatment group were evaluated using independent-samples *t*-tests, with statistical significance defined at *p* < 0.05. Data visualization was performed using GraphPad Prism 8.0 software.

## 3. Results

### 3.1. Quality Assessment of Transcriptome Sequencing and Assembly

Transcriptome sequencing was performed on 42 cDNA libraries derived from the gill and hepatopancreas tissues of *S. constricta* exposed to ammonia, hypoxia, and combined stress. After rigorous quality control, a total of 165.80 Gb of clean data was obtained, with 90.72 Gb from gill samples and 75.08 Gb from hepatopancreas samples. The sequencing quality metrics were high, with Q30 percentages ranging from 92.96% to 95.57% (average 92.96%) and a consistent GC content of approximately 39.17% (Table 2). Sequence alignment against the *S. constricta* reference genome yielded mapping rates between 72.1% and 81.48%. These metrics confirmed that the sequencing data were of sufficient quality and depth for subsequent differential expression and network analyses.

### 3.2. Divergent Temporal and Tissue-Specific Differential Expression

**Figure 1:**
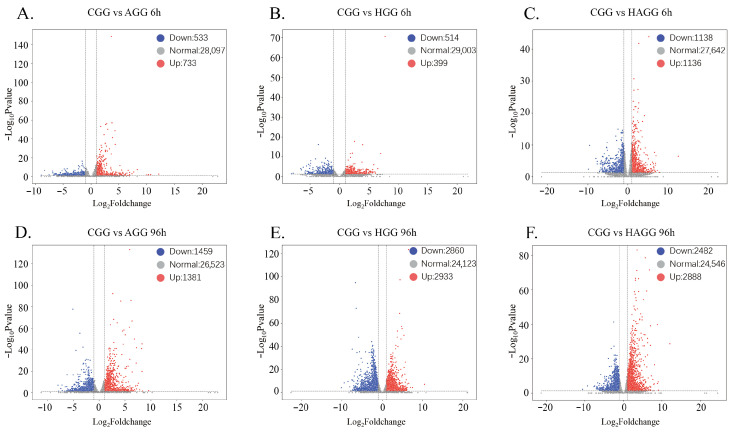
Volcano plots of differentially expressed genes (DEGs) in the gill of *S. constricta* under different stress conditions. (**A**–**C**) Pairwise comparisons at 6 h for AG, HG, and HAG groups versus control (CGG). (**D**–**F**) Pairwise comparisons at 96 h. Red dots indicate upregulated genes; blue dots indicate downregulated genes.

**Figure 2:**
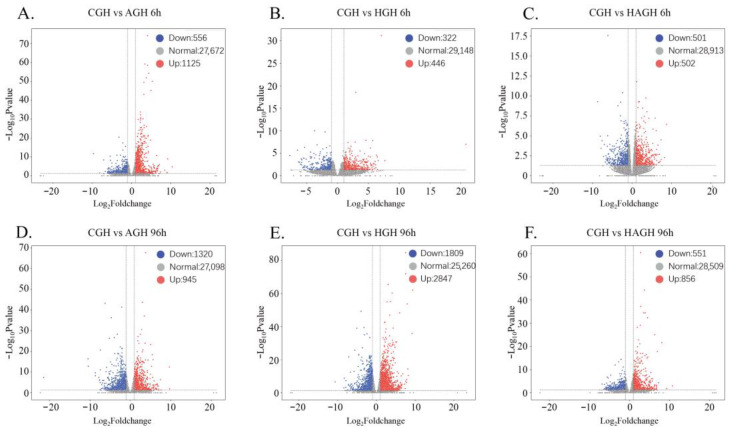
Volcano plots of differentially expressed genes (DEGs) in the gill of *S. constricta* under different stress conditions. (**A**–**C**) Pairwise comparisons at 6 h for AG, HG, and HAG groups versus control (CGH). (**D**–**F**) Pairwise comparisons at 96 h. Red dots indicate upregulated genes; blue dots indicate downregulated genes.

Differential expression analysis revealed distinct temporal and tissue-specific response patterns under ammonia (AG), hypoxia (HG), and combined stress (HAG) (Figure 1 and Figure 2).

In the gill, the transcriptional response was characterized by a progressive recruitment of responsive genes over time. Under AG stress, the number of differentially expressed genes (DEGs) more than doubled from 1266 (733 upregulated and 533 downregulated) at 6 h to 2840 (1381 upregulated and 1459 downregulated) at 96 h (Figure 1A,D). Similarly, HG stress induced 913 DEGs (399 upregulated and 514 downregulated) at 6 h, which increased markedly to 5793 DEGs (2933 upregulated and 2860 downregulated) at 96 h (Figure 1B,E). Notably, the combined stress (HAG) elicited the most extensive transcriptional changes including 2274 DEGs (1136 upregulated and 1138 downregulated) at 6 h, rising to 5370 DEGs (2888 upregulated and 2482 downregulated) at 96 h (Figure 1C,F). This trajectory indicates a sustained and intensifying physiological burden on the respiratory organ.

In the hepatopancreas, the temporal dynamics differed significantly. While AG and HG exposure increased the number of DEGs over time, which reached 2265 (945 upregulated and 1320 downregulated) (Figure 2A,D) and 4656 (2847 upregulated and 1809 downregulated) (Figure 2B,E) at 96 h, respectively, the combined stress (HAG) resulted in a comparatively dampened response at 96 h (1416 DEGs containing 856 upregulated and 551 downregulated) relative to the single stressors (Figure 2C,F). This reduction in transcriptional activity suggests a shift toward metabolic depression under prolonged dual stress.

### 3.3. Functional Enrichment and Pathway Analysis

**Figure 3:**
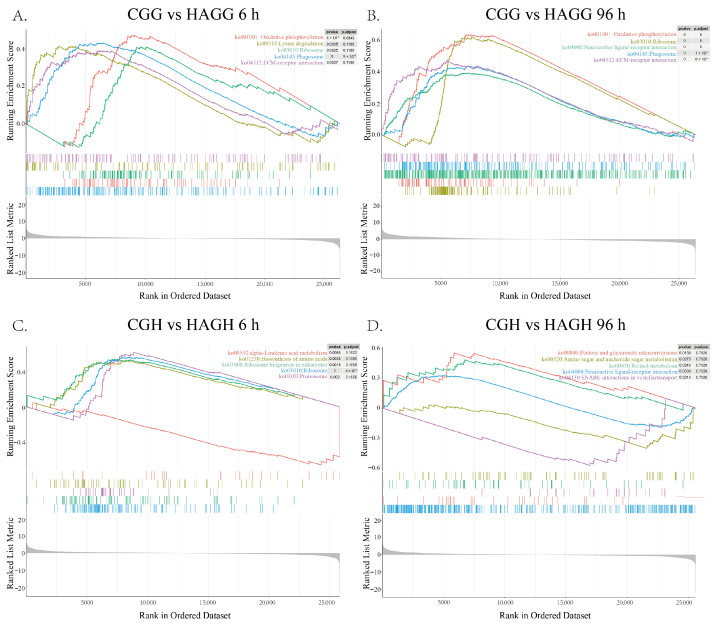
Gene set enrichment analysis (GSEA) of key pathways. (**A**,**B**) Enriched pathways in the gill under HAG stress at 6 h and 96 h relative to control. (**C**,**D**) Enriched pathways in the hepatopancreas under HAG stress. Curves above the zero line indicate activation; curves underneath this line indicate suppression.

**Figure A2:**
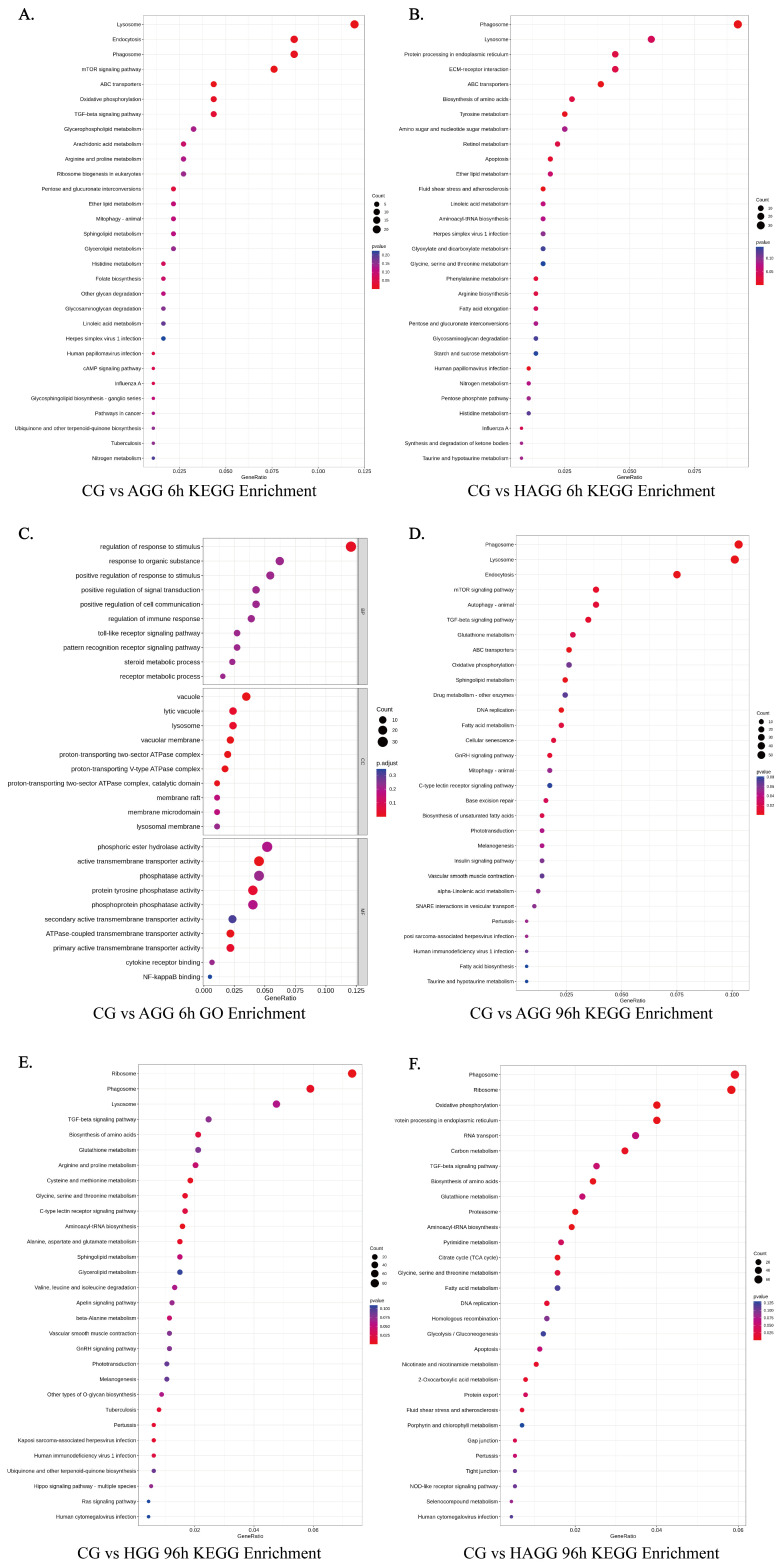
KEGG and GO enrichment in gill of *S. constricta* under different stress conditions. (**A**) KEGG enrichment at 6 h for AG. (**B**) KEGG enrichment at 6 h for HAG. (**C**) GO enrichment at 6 h for AG. (**D**) KEGG enrichment at 96 h for AG. (**E**) KEGG enrichment at 96 h for HG. (**F**) KEGG enrichment at 96 h for HAG.

**Figure A3:**
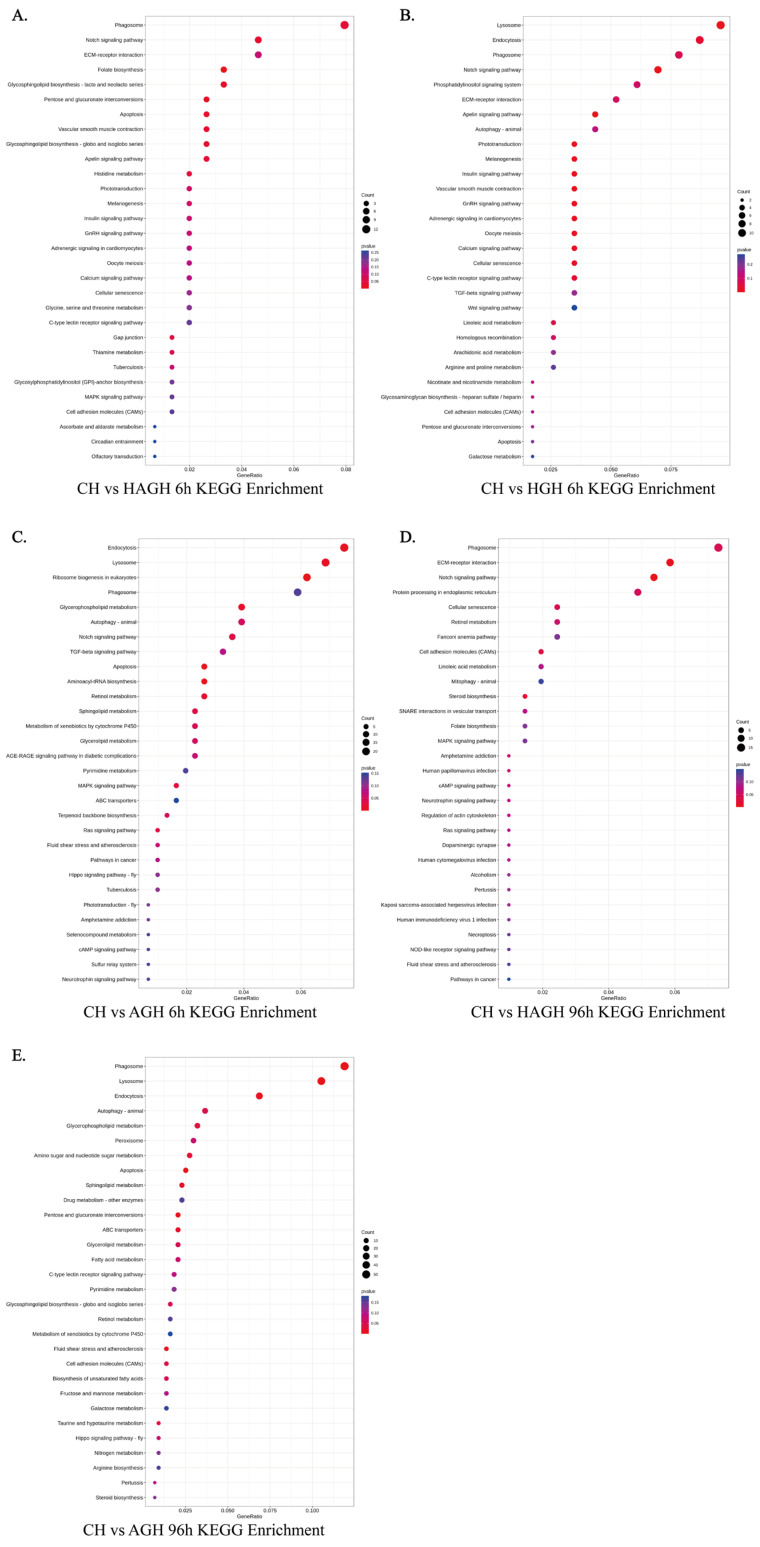
KEGG and GO enrichment in hepatopancreas of *S. constricta* under different stress conditions. (**A**) KEGG enrichment at 6 h for HAG. (**B**) KEGG enrichment at 6 h for HG. (**C**) KEGG enrichment at 6 h for AG. (**D**) KEGG enrichment at 96 h for HAG. (**E**) KEGG enrichment at 96 h for AG.

**Figure A4:**
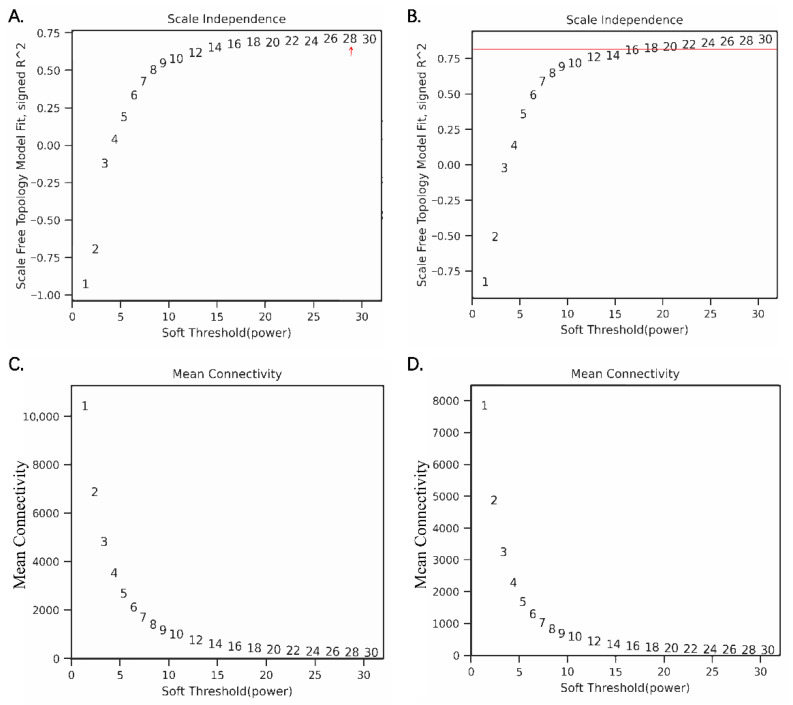
Determination of soft-thresholding power for WGCNA network construction. (**A**) Scale-free topology model fit (signed R^2^) as a function of soft-thresholding power (β) in gill tissue. The red arrow indicates the attainment of a plateau, R^2^ = 0.7. (**B**) Scale-free topology model fit as a function of soft-thresholding power in hepatopancreas tissue. The red line indicates the standard threshold. R^2^ = 0.8. (**C**) Mean connectivity as a function of soft-thresholding power in gill tissue. (**D**) Mean connectivity as a function of soft-thresholding power in hepatopancreas tissue. The numerical values indicated in the figure correspond to the soft thresholds located on the horizontal axis.

To elucidate the biological functions underlying these transcriptional changes, KEGG and GO enrichment analyses, along with GSEA, were performed (Appendix A Figure A2 and Figure A3 and Figure 3). These analyses revealed that the gill and hepatopancreas employ fundamentally different molecular strategies to cope with stress.

#### 3.3.1. Gill Responses: From Immune Defense to Bioenergetic Remodeling

In the gill, under AG and HAG stress (6 h), the transcriptome was dominated by cellular quality control and immune signaling. Significant enrichment was observed in “Lysosome”, “Endocytosis”, and “mTOR signaling” pathways (Appendix A Figure A2A,B). GO analysis further highlighted the activation of innate immune responses, specifically “Toll-like receptor (TLR) signaling” and “Pattern recognition receptor activity”, alongside “V-type ATPase-mediated acidification”, suggesting an immediate mobilization of mucosal immunity and pH regulation (Appendix A Figure A2C).

As stress persisted for 96 h, the functional landscape shifted. After AG stress at 96 h, the gill prioritized “Lipid metabolism remodeling” and “ABC transporter-mediated detoxification” (Appendix A Figure A2D). However, under HG and HAG conditions, massive bioenergetic and structural reprogramming occurred (Appendix A Figure A2E,F). The KEGG and GSEA results concurrently identified a robust upregulation of “Oxidative phosphorylation” and the “Citrate cycle (TCA cycle)” (Figure 3A), underscoring a compensatory effort to sustain ATP production. This was accompanied by a synergistic enhancement of protein turnover machinery, evidenced by the enrichment of “Ribosome biogenesis”, “Proteasome” and “Ribonucleoprotein complex” terms (Figure 3B). These profiles indicate that the gill attempts to maintain high metabolic activity and structural integrity despite the energy limitations imposed by hypoxia.

#### 3.3.2. Hepatopancreas Responses: Metabolic Reprogramming and Stress Damage

The hepatopancreas displayed a response centered on metabolic flexibility and detoxification, with signs of physiological strain appearing under combined stress. The early response involved rapid signaling adjustments when exposure for 6 h (Appendix A Figure A3A). Under hypoxia, pathways related to “Notch signaling” and “Calcium signaling” were enriched, suggesting an early sensing mechanism (Appendix A Figure A3B). Concurrently, acute ammonia exposure triggered the enrichment of “Cytochrome P450” and “Drug metabolism” pathways, highlighting the organ’s role in chemical detoxification (Appendix A Figure A3C). Furthermore, the most critical response occurred at 96 h, particularly under HAG stress (Appendix A Figure A3D). Functional analysis revealed a strategic pivot in nitrogen handling. Pathways for “Arginine biosynthesis”, “Nitrogen metabolism” and “Alanine, aspartate and glutamate metabolism” were significantly modulated (Appendix A Figure A3E). GSEA specifically highlighted shifts in “Urea cycle” intermediates and “Polyamines”, supporting a redirection of arginine flux.

However, distinct from the gill, the hepatopancreas showed molecular signatures of severe stress overload under chronic HAG conditions. Enriched terms included “Protein processing in endoplasmic reticulum (ER stress)”, “Cellular senescence” and “Steroid biosynthesis” (Appendix A Figure A3D). GSEA further identified perturbations in “Pentose and glucuronate interconversions” and “Retinol metabolism” (Figure 3D). The transition from early signaling regulation to late-stage senescence and ER stress signatures suggests that the hepatopancreas faces a “metabolic ceiling” and accumulated cellular damage under prolonged dual stress.

### 3.4. Identification of Stress-Responsive Gene Modules via WGCNA

To unravel the co-expression dynamics in response to ammonia and hypoxic stress, weighted gene co-expression network analysis (WGCNA) was independently performed on 21 samples from the gill and hepatopancreas of *S. constricta*.

**Figure 4:**
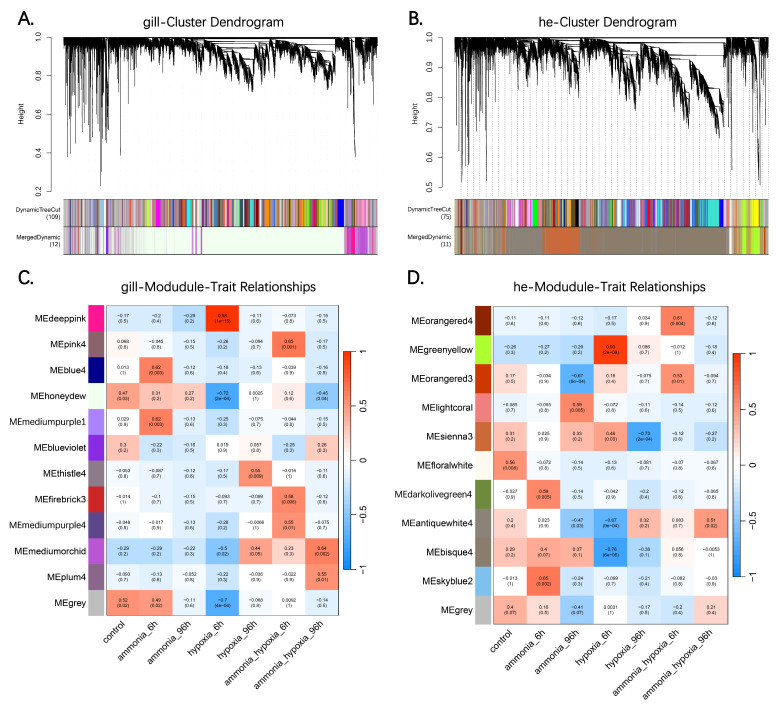
WGCNA of *S. constricta* transcriptomes. (**A**,**B**) Clustering dendrograms of gill and hepatopancreas. (**C**,**D**) Module–trait relationship heatmaps. The color scale represented the Pearson correlation coefficient (red: positive; green: negative). Modules with *p* < 0.05 were considered significantly associated with specific traits.

For the gill tissue, a scale-free network was constructed using an optimized soft-thresholding power of *β* = 28 (R^2^ > 0.8). A total of 12 distinct co-expression modules were identified, with gene numbers ranging from 26 to 12,694 (Figure 4A; Table 3). Correlation analysis revealed that the mediumorchid module (1233 genes) exhibited a strong positive correlation with HAG stress (r = 0.64, *p* = 0.002) (Figure 4C), suggesting its specific involvement in the synergistic response. Furthermore, the honeydew module, which represents the largest gene cluster (12,694 genes), was examined for its biological relevance. Although its correlation with stress traits was moderate (r = 0.31, *p* = 0.2), this module was found to contain the critical oxygen-sensing regulators *HIF1-α* and *HIF-2α*, indicating that the basal oxygen-sensing machinery resides within this core network.

Similarly, the hepatopancreas network was established with *β* = 20. Eleven modules containing between 50 and 6822 genes were defined, revealing a highly specific response pattern to acute hypoxia (Figure 4B; Table 3). Two key modules displayed strong but opposing correlations with the acute hypoxia (6 h) and combined stress traits (Figure 4D). The greenyellow (774 genes) showed a highly significant positive correlation (r = 0.93, *p* = 1.79 × 10^−9^). Functional screening identified *ARG* as a high-connectivity hub gene within this module, consistent with its upregulation under stress. The *bisque4* modules (6822 genes) showed a significant negative correlation (r = −0.76, *p* = 6.01 × 10^−5^). Notably, *NOS* was mapped to this module. The distinct compartmentalization of *ARG* (positive module) and *NOS* (negative module) in the hepatopancreas co-expression network provides statistical support for their reciprocal regulation, pointing to a coordinated metabolic shift towards the urea cycle and away from nitric oxide synthesis under early stress conditions.

**Figure 5:**
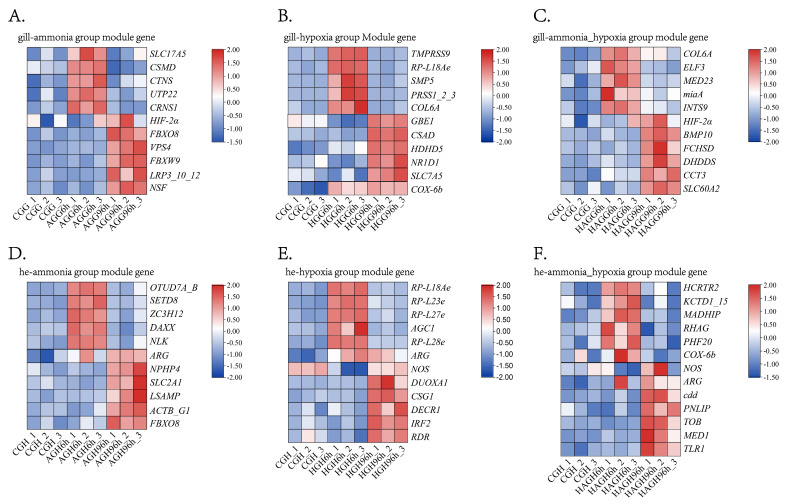
Hierarchical clustering heatmap of representative hub genes identified via WGCNA. The heatmap displays gene expression levels based on log_10_(FPKM + 1) values. (**A**) Genes from modules MEhoneydew_ammonia_6h (*SLC17A5*, *CSMD*, *CTNS*, *UTP22*, *CRNS1*, and *HIF-2α*) and MEhoneydew_ammonia_96h (*FBXO8*, *VPS4*, *FBXW9*, *LRP3_10_12*, and *NSF*). (**B**) Genes belonging to modules MEdeeppink_hypoxia_6h (*TMPRSS9*, *RP-L18Ae*, *SMP5*, *PRSS1_2_3*, and *COL6A*), and MEmediumorchid_hypoxia_96h (*GBE1*, *CSAD*, *HDHD5*, *NR1D1*, *SLC7A5*, and *COX-6b*). (**C**) Candidates from modules MEmediumorchid_ammonia_hypoxia_6h (*COL6A*, *ELF3*, *MED23*, *miaA*, and *INTS9*), MEhoneydew_ammonia_hypoxia_96h (*HIF-2α*), and MEmediumorchid_ammonia_hypoxia_96h (*BMP10*, *FCHSD*, *DHDDS*, *CCT3*, and *SLC60A2*). (**D**) Genes identified from modules MEbisque4 ammonia_6h (*OTUD7A_B*, *SETD8*, *ZC3H12*, *DAXX*, and *NLK*), Megreenyellow ammonia_6h/96h (*ARG*), and MEbisque4 ammonia_96h (*NPHP4*, *SLC2A1*, *LSAMP*, *ACTB_G1* and *FBXO8*). (**E**) Genes from modules Megreenyellow hypoxia_6h (*RP-L18Ae*, *RP-L23e*, *RP-L27e*, *AGC1*, and *RP-L28e*), Mebisque4 hypoxia_6h/96h (*ARG* and *NOS*), and Meantiquewhite4 hypoxia_96h (*DUOXA1*, *CSG1*, *DECR1*, *IRF2*, and *RDR*). (**F**) Integrated expression of key markers from modules MEorangered4_hypoxia_6h (*HCRTR2*, *KCTD1_15*, *MADHIP*, *RHAG*, and *PHF20*), MEbisque4_hypoxia_6h (*NOS*), MEgreenyellow_ammonia_hypoxia_6h/96h (*COX-6b*), MEbisque4_hypoxia_96h (*ARG*), and MEantiquewhite4_hypoxia_96h (*cdd*, *PNLIP*, *TOB*, *MED1*, and *TLR1*).

To further evaluate the expression patterns of key regulatory candidates, a heatmap was constructed based on the log_10_ (FPKM+1) values of representative genes (Figure 5). The selection prioritized genes from the module exhibiting the highest correlation with the target traits, provided that consistent expression profiles were maintained across biological replicates. Specifically, the top five annotated genes with the highest intramodular connectivity within the *S. constricta* genomic database were selected, alongside several critical physiological markers, including *HIF-1α*, *HIF-2α*, *COX-6b*, *ARG*, and *NOS*. Hierarchical clustering analysis revealed that these genes exhibited distinct and cohesive expression profiles across different experimental groups, indicating robust modularity and high data reliability for subsequent functional analysis.

### 3.5. qRT-PCR Validation of Key Genes

In the gill tissues, the different *HIF* isoforms showed divergent expression dynamics. Specifically, *HIF-1α* mRNA levels were consistently downregulated across all stress groups relative to the control. In contrast, *HIF-2α* displayed a time-dependent response. It was downregulated during the early stages (6 h) of ammonia and hypoxic stress but significantly upregulated during prolonged exposure (96 h) to hypoxia and combined stress. Additionally, the mitochondrial gene *COX-6b* was exclusively upregulated under hypoxic conditions (HG and HAG) at both time points of 6 h and 96 h.

**Figure 6:**
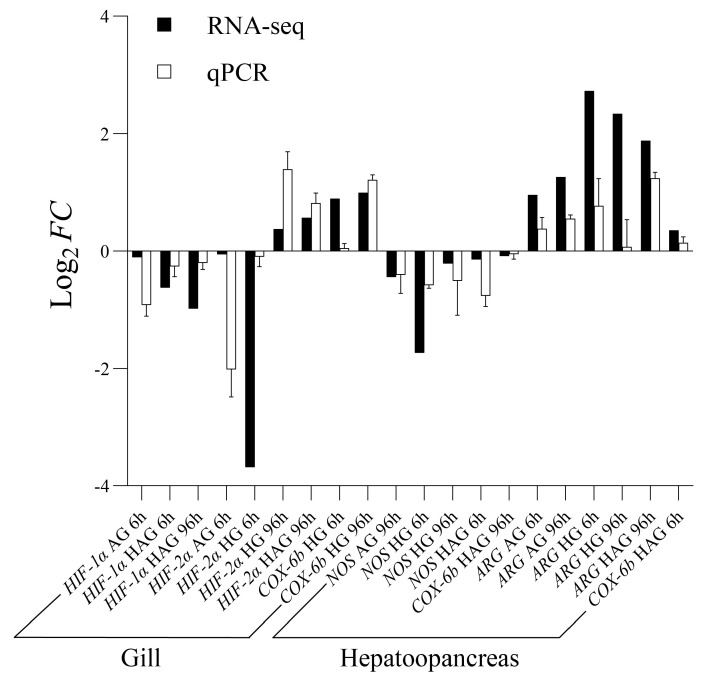
Validation of RNA-seq data via qRT-PCR. Comparison of expression levels for key genes in the gill (*HIF-1α*, *HIF-2α*, *COX-6b*) and hepatopancreas (*ARG*, *NOS*, *COX-6b*) of *S. constricta*. Black bars indicate transcriptomic data (FPKM), and white bars represent qRT-PCR data (2^−△△Ct^). Data are presented as mean ± SEM (*n* = 3).

In the hepatopancreas, genes involved in arginine metabolism showed reciprocal regulation. *Arginase* (*ARG*) exhibited a robust and consistent upregulation across nearly all stressed groups at 6 h and 96 h. Conversely, *NOS* was predominantly downregulated, particularly under combined stress at 96 h. The expression of *COX-6b* in the hepatopancreas differed from that in the gill, showing a transient increase at 6 h in HAG group followed by a significant decline at 96 h. Overall, the qRT-PCR results demonstrated a high degree of concordance with the RNA-seq data, confirming the reliability of the sequencing results (Figure 6).

## 4. Discussion

The escalating prevalence of coastal eutrophication and global warming—two interconnected processes that severely alter marine chemistry—has rendered the co-occurrence of ammonia nitrogen and hypoxia a frequent environmental challenge in aquaculture ecosystems [6,26]. Coastal eutrophication occurs when excess nutrients, primarily nitrogen and phosphorus from agricultural runoff and wastewater, enter the ocean [27,28]. These nutrients act as fertilizers, triggering massive blooms of algae [29]. As the algae eventually die and sink, their decomposition by bacteria consumes vast amounts of dissolved oxygen, leading to hypoxia (low oxygen). Simultaneously, the breakdown of organic matter and the excretion from dense aquaculture populations release high levels of ammonia [30]. Consequently, eutrophication creates a ‘double threat’ where marine organisms must simultaneously cope with toxic ammonia levels and a suffocating lack of oxygen. While the independent effects of these stressors have been extensively mapped in aquatic invertebrates, high-throughput transcriptomics has only recently begun to characterize the molecular responses in mollusks [31,32,33]. This study provides a comprehensive analysis of the combined environmental stress response in the benthic bivalve *S. constricta*. Our integrated WGCNA and functional enrichment analyses reveal that this species employs distinct tissue-specific physiological strategies rather than a uniform systemic response. The gill primarily modulates oxygen sensing and respiratory efficiency, while the hepatopancreas executes a significant metabolic reprogramming centered on arginine metabolism. These findings highlight the critical physiological trade-offs required to survive the combined burden of toxicity and oxygen limitation, a pattern broadly supported by recent multi-omics studies in aquatic ectotherms [34,35].

### 4.1. Differential Regulation of Oxygen Sensing and Respiratory Remodeling in Gills

The gill acts as the primary interface for gas exchange and the first line of defense against environmental stressors, making it highly susceptible to tissue damage and inflammation [11,36]. A central finding of this study was the divergent temporal regulation of HIF isoforms. Classically, *HIF-1α* is regarded as the master regulator of oxygen homeostasis, stabilizing during acute hypoxia to drive glycolytic gene expression [37]. However, we observed a consistent transcriptional downregulation of *HIF-1α* mRNA across the ammonia and combined stress groups as early as 6 h. This suggests that ammonia nitrogen may interfere with the classical hypoxic signaling pathway during the acute response phase. This apparent contradiction underscores the distinction between transcriptional and post-translational control. While HIF-1α protein stabilization is essential for acute survival, sustained overexpression of *HIF-1α* mRNA has been linked to excessive inflammation and tissue damage in aquatic invertebrates, as recently demonstrated in *Litopenaeus vannamei* [38] and other crustaceans [39]. Furthermore, comparative studies in shrimp have shown that *HIF-1α* expression patterns can vary significantly depending on the stress duration and genetic background [40]. As demonstrated in previous studies, stress conditions can selectively increase the expression of HIF-1 inhibitors, such as prolyl hydroxylases (PHDs), which act as a negative feedback mechanism to modulate the hypoxic response [41]. Therefore, we postulate that the observed early transcriptional suppression in *S. constricta* likely represents a rapid negative feedback response or a direct inhibitory effect of ammonia toxicity, aimed at preventing the premature accumulation of pro-apoptotic factors and mitigating cellular damage.

In contrast, *HIF-2α* was significantly upregulated during the chronic phase (96 h) of hypoxia and combined stress. In mammalian models and recent bivalve studies, *HIF-2α* is often implicated in chronic adaptation, governing the expression of genes involved in structural maintenance and erythropoiesis, distinct from the acute glycolytic role of *HIF-1α* [23]. The specific recruitment of *HIF-2α* under combined stress suggests a shift in regulatory dominance, facilitating a transition from an acute emergency response to a long-term homeostatic state that allows the gill to adapt to persistent oxygen limitation [13].

Downstream of this oxygen sensing, the upregulation of *cytochrome c oxidase subunit 6b* (*COX-6b*) in gills under hypoxic conditions (HG and HAG) indicates a targeted remodeling of the mitochondrial electron transport chain (ETC). As the terminal enzyme of the ETC, COX efficiency determines the rate of ATP production. Previous investigations on the bivalve *Arctica islandica* have shown that modulating NO levels and COX activity is a key strategy to match metabolic demand with oxygen supply [15]. By upregulating *COX-6b*, *S. constricta* gills attempt to maximize oxygen affinity to support cilia beating and osmoregulation. However, this compensatory effort appears to come at a physiological cost. The concurrent enrichment of “Apoptosis” and “NOD-like receptor signaling” pathways in our data suggest that maintaining high respiratory drive under stress generates excessive reactive oxygen species (ROS), leading to inflammatory damage [42,43]. Thus, the gill strategy involves a delicate trade-off between maintaining oxygen uptake and managing oxidative injury.

### 4.2. Reciprocal Regulation of Arginine Metabolism in the Hepatopancreas

The hepatopancreas serves as the metabolic hub for detoxification and energy storage, playing an essential role in the response to ammonia toxicity [21,44]. Our study identified arginine metabolism as the core adaptive module in this tissue, driven by the reciprocal regulation of ARG and NOS. L-arginine acts as the common substrate for both ARG (producing urea and ornithine) and NOS (producing nitric oxide) [12]. In our dataset, *ARG* was universally upregulated while *NOS* was downregulated. This expression signature indicates that *S. constricta* actively redirects arginine flux towards the urea/ornithine cycle. Although bivalves are ammonotelic, the upregulation of *ARG* suggests that its functional role is not limited to simple nitrogen excretion but may also be involved in polyamine biosynthesis or amine clearance. Regarding polyamines biosynthesis, ornithine (the product of arginase) serves as a precursor for polyamines (putrescine, spermidine), which are essential for DNA repair and membrane stabilization. Comparative studies in teleosts have shown that enhancing the ornithine pathway can significantly alleviate ammonia-induced oxidative stress [45]. It is likely that *S. constricta* employs a similar mechanism to protect hepatopancreatic cells from ammonia toxicity. Furthermore, ammonia clearance is facilitated by converting the excess endogenous and exogenous ammonia in the body into urea, thereby reducing the burden of free, non-ionized ammonia (NH_3_) in the blood and lymph of the organism [14]. This aligns with findings in other bivalves where urea cycle intermediates are modulated to buffer nitrogen overload [34].

Significantly, the downregulation of *NOS* reveals a vital bioenergetic adaptation to the combined stressors. Nitric oxide (NO) is a potent reversible inhibitor of cytochrome c oxidase (COX, Complex IV), the terminal enzyme of the mitochondrial electron transport chain. NO competes directly with O_2_ for the reduced binuclear center of *COX* [46]. Under normoxia, this interaction serves as a physiological rheostat for metabolic rate; however, under hypoxia, the decreased O_2_ availability significantly enhances the inhibitory potency of NO, potentially leading to a total blockade of mitochondrial respiration. Strahl and Abele [15] demonstrated in *Arctica islandica* that maintaining low NO levels is a prerequisite for sustaining mitochondrial function during environmental challenges. By suppressing *NOS* expression, the hepatopancreas of *S. constricta* effectively alleviates this NO-mediated respiratory inhibition. This adjustment ensures that the limited O_2_ molecules can be utilized by *COX* for ATP generation rather than being excluded by NO. Furthermore, reducing NO production limits its reaction with superoxide anions to form peroxynitrite (ONOO^−^), thereby preventing the accumulation of this highly reactive nitrogen species and subsequent oxidative damage [47].

However, this strategy carries an immunological risk. NO is a key antimicrobial effector in invertebrate innate immunity. The suppression of *NOS* to conserve energy and reduce toxicity may render *S. constricta* more susceptible to opportunistic pathogens, consistent with the immune suppression and autophagy dysregulation observed in other aquatic species under ammonia stress [6,48].

### 4.3. Mechanisms of Synergistic Toxicity and Metabolic Depression

Our transcriptomic profiles clearly demonstrate that combined stress (HAG) exerts a synergistic, rather than merely additive, negative impact, confirming observations in other aquatic species such as shrimp [16] and fish [19]. This is most evident in the transcriptome suppression observed in the hepatopancreas at 96 h, where the number of DEGs was lower than in single stress groups. This phenomenon is indicative of metabolic depression, a well-documented survival strategy in benthic invertebrates where ATP turnover is actively suppressed to extend survival time during environmental extremes [15].

We propose a bioenergetic limitation hypothesis to explain this synergistic toxicity. Ammonia detoxification is an ATP-dependent process. For instance, the conversion of glutamate to glutamine by glutamine synthetase (GS) requires ATP [12], and the maintenance of ion gradients relies on Na^+^/K^+^-ATPase. Under AG stress, the organism upregulates these pathways [49]. However, hypoxia imposes a severe ceiling on aerobic ATP production. When combined (HAG), the energy demand for detoxification exceeds the supply limited by hypoxia [20].

This ATP supply–demand mismatch forces the hepatopancreas into a state of metabolic depression, characterized by the global downregulation of biosynthetic pathways (e.g., “Ribosome biogenesis”, “Amino acid biosynthesis”) observed in our GSEA results. While metabolic depression extends survival, it halts repair processes [9]. The enrichment of “Phagosome” and “ECM–receptor interaction” pathways at HAG 96 h indicates that structural damage accumulates faster than it can be repaired, leading to tissue remodeling [50]. This accumulation of damage explains why the combined stressors are lethal even at concentrations that are sublethal individually [18].

Despite these insights, several limitations should be acknowledged. First, the sample scope of this study was focused on specific tissues (gill and hepatopancreas) under controlled laboratory conditions; however, the physiological responses might exhibit greater complexity in wild populations across different life stages. Second, we did not fully account for environmental externalities, such as fluctuating temperatures or co-existing pollutants in the natural benthos, which could synergistically influence nitrogen metabolism. Furthermore, our reliance on transcriptomic data means that post-translational modifications (e.g., *HIF-1α* protein stability) and actual enzymatic activities remain to be verified.

Future research should integrate multi-omics approaches, such as metabolomics, to confirm the flux of arginine towards polyamines [51]. Additionally, investigating the role of sediment microbiota in nitrogen cycling [8] and conducting field-based studies will be essential to validate these laboratory findings in more ecologically relevant contexts.

## 5. Conclusions

In summary, the resilience of *S. constricta* to ammonia and hypoxia stems from a dual-layered adaptive framework. On an evolutionary scale, the species has developed fixed, tissue-specific mechanisms—such as the specialized oxygen-sensing switch in the gill—that reflect its long-term commitment to the sessile, benthic lifestyle. Complementing these innate traits is a flexible range of individual physiological adaptations, where clams dynamically reprogram their metabolic pathways, such as prioritizing ammonia detoxification over immune signaling, to survive inevitable and fluctuating environmental shifts. The organism navigates this environmental challenge by uncoupling the responses of its respiratory and metabolic organs. The gill prioritizes oxygen uptake through *COX-6b* upregulation and differential *HIF* expression, while the hepatopancreas adopts a protective state characterized by reduced NO synthesis and enhanced urea cycle activity. Understanding these tissue-specific limitations provides a theoretical foundation for developing stress-resilient strains for sustainable aquaculture.

## Figures and Tables

**Table 1:** Primer information of genes detected by qRT-PCR.

Gene	Forward Primer	Reverse Primer	Tm	Product Size (bp)
*HIF-1α*	5′ TTTCCTGGTCGTTCTCTCCAA 3′	5′ TCACGCTCCTGCCCTTACTG 3′	58 °C	248
*HIF-2α*	5′ CTTTTGAGCAGAACTTTCAGACAG 3′	5′ TCGCACTCTCATATCCACCATC 3′	58 °C	247
*ARG*	5′ GGAGTGTGTAGAGAGAGCGATG 3′	5′ TGCTGGGAATGTATGTGGG 3′	56 °C	104
*NOS*	5′ CGGACACATTCAGGGTCACA 3′	5′ GCGCGTTATGAAGACGCATT 3′	57 °C	273
*COX-6b*	5′ AACAGAAGCGTGAGAATGACC 3′	5′ CAGATAGAAGCTGGGGCAG 3′	59 °C	221
*RS9*	5′ TGAAGTCTGGCGTGTCAAGT 3′	5′ CGTCTCAAAAGGGCATTACC 3′	59 °C	117

**Table 2:** Summary of RNA-seq data quality and alignment statistics for *S. constricta* gill and hepatopancreas tissues.

Sample	Raw_Reads_Num	Raw_Bases (G)	Clean_Reads_Num	Clean_Bases (G)	Clean_Rate (%)	Q20 (%)	Q30 (%)	GC (%)	Mapping_Rate (%)
CGG	108,853,368	16.33	108,826,303	16.23	99.97	98.16	94.15	39.22	72.10%
AGG 6h	71,803,806	10.77	71,787,926	10.7	99.98	98.21	94.27	39.17	72.22%
AGG 96h	95,484,023	14.32	95,462,808	14.21	99.98	98.09	94.06	40.26	73.90%
HGG 6h	62,716,249	9.41	62,692,322	9.36	99.96	97.88	93.99	40.69	74.05%
HGG 96h	95,009,534	14.25	94,978,727	14.15	99.97	98.32	95.23	42.22	77.72%
HAGG 6h	79,587,504	11.94	79,562,041	11.85	99.97	98.28	95.11	42.39	76.78%
HAGG 96h	95,575,913	14.33	95,545,385	14.22	99.97	98.42	95.4	43.14	79.71%
CGH	71,608,053	10.74	71,579,899	10.66	99.96	97.64	92.96	43.64	77.36%
AGH 6h	65,354,338	9.80	65,330,031	9.72	99.96	97.66	92.98	43.66	77.85%
AGH 96h	73,479,669	11.02	73,450,055	10.92	99.96	97.93	93.71	43.69	78.68%
HGH 6h	63,360,131	9.50	63,328,545	9.43	99.95	97.82	93.94	42.85	75.21%
HGH 96h	84,669,488	12.70	84,624,757	12.58	99.95	98.47	95.57	45.47	81.48%
HAGH 6h	86,758,469	13.01	86,721,155	12.91	99.96	98.44	95.35	44.95	79.46%
HAGH 96h	59,617,105	8.94	59,588,146	8.86	99.95	98.48	95.44	44.84	79.67%

Abbreviations: CGG and CGH, gill and hepatopancreas under control group (CG); AGG and AGH, gill and hepatopancreas under ammonia nitrogen (AG) stress; HGG and HGH, gill and hepatopancreas under hypoxia (HG) stress; HAGG and HAGH, gill and hepatopancreas under combined (HAG) stress.

**Table 3:** Gene numbers in different modules obtained by a WGCNA.

Modules (Gill)	Counts (Gill)	Modules (He)	Counts (He)
honeydew	12,694	bisque4	6822
gray	2148	antiquewhite4	2647
mediumorchid	1233	sienna3	1926
deeppink	471	gray	930
blueviolet	167	greenyellow	774
mediumpurple4	74	orangered3	188
pink4	73	orangered4	147
mediumpurple1	64	floralwhite	134
firebrick3	49	skyblue2	77
blue4	42	lightcoral	62
plum4	34	darkolivegreen4	50
thistle4	26		

## Data Availability

Raw transcriptome data are available in the NCBI SRA under accession number: PRJNA1420959.
